# Functional Effects of *Bacillus velezensis* Metabolites on Barrier Formation, Cytokine Responses, and Phagocytic Activity in Canine Epithelial and Immune Cells

**DOI:** 10.3390/ijms27104417

**Published:** 2026-05-15

**Authors:** Andreea Cornelia Udrea, Katrine Bie Larsen, Akila Rekima, Adrian Schwarzenberg, Steffen Yde Bak, Niels Christensen, Chong Shen

**Affiliations:** 1 Gut Immunology Lab, Research and Development, Health & Biosciences, IFF, Edwin Rahrs Vej 38, 8220 Aarhus, Denmark; andreea.cornelia.udrea@iff.com (A.C.U.); katrinebie.larsen@iff.com (K.B.L.); akila.rekima@iff.com (A.R.); 2 Enabling Technologies, Research and Development, Health & Biosciences, IFF, Edwin Rahrs Vej 38, 8220 Aarhus, Denmark; adrian.schwarzenberg@iff.com (A.S.); steffen.yde.bak@iff.com (S.Y.B.); niels.christensen@iff.com (N.C.)

**Keywords:** *Bacillus velezensis*, probiotic consortia, canine proximal gastrointestinal epithelium, epithelial barrier function, cytokine regulation, macrophage activation, epithelial renewal, metabolomics, redox homeostasis

## Abstract

*Bacillus velezensis*-based probiotics are increasingly recognized for their potential to enhance intestinal health in companion animals, yet their mechanisms of action in canine epithelial systems remain incompletely defined. This study aimed to evaluate whether a live *Bacillus velezensis* probiotic consortia (BC) modulates epithelial barrier integrity, immune signaling, apoptosis-renewal pathways, and metabolic activity in canine-relevant intestinal and macrophage cell models. MCA-B1 proximal gastrointestinal epithelial cells and DH82 macrophage-like cells were exposed to BC cultures, followed by quantification of tight-junction expression, permeability (FITC-Dextran), cytokine responses, phagocytic activity, apoptosis-related markers, and metabolomic profiles. BC treatment significantly strengthened the epithelial barrier, inducing a marked upregulation of Claudin 1 (CLDN1) (11.3 fold), CLDN4 (2.4 fold), Occludin (OCLN, 1.7 fold), and increasing key proteins including ZO-2 and cingulin while reducing LPS-induced FITC-Dextran permeability to 94.5%. BC concurrently modulated innate immune signaling, increasing MyD88 (33.2%), IL-8 (14.6 fold), IL-18 (2.6 fold), and IFNB1 protein levels, while enhancing anti-inflammatory regulation, including a robust rise in DH82-derived IL-10. Apoptosis-renewal markers shifted toward physiological turnover, with increased BCL2 (1.9 fold) and reduced BAK1. Metabolomic profiling of BC activity revealed elevated AMP, abundant Peptide Transporter 1 (PEPT1)-transportable peptides, increased γ-glutamyl metabolites, and lower Glutathione disulfide (GSSG), consistent with AMPK-linked tight-junction assembly and glutathione-supported redox buffering. Together, these data indicate that *Bacillus velezensis*-derived metabolites positively influence barrier-related, immunological, and metabolic responses in a canine proximal intestinal epithelial system and modulate functional responses in macrophage-like cells. These in vitro findings contribute to the mechanistic understanding of host cellular responses to *Bacillus*-associated metabolites.

## 1. Introduction

The gastrointestinal (GI) tract plays a central role in canine health, governing nutrient absorption, mucosal immunity, and metabolic and behavioral homeostasis. Disturbances caused by diet changes, antibiotics, stress, or underlying disease can disrupt microbial balance and impair epithelial barrier function, contributing to acute diarrhea and chronic enteropathies. As interest grows in nutritional strategies to support GI resilience, probiotics—particularly spore-forming *Bacillus* species—have gained attention due to their stability and immunomodulatory potential. Recent reviews underscore the importance of the canine gut microbiome in immune regulation and metabolic signaling, and highlight opportunities to support GI health through targeted microbial interventions [1,2].

Several peer reviewed studies have evaluated *Bacillus* spp. in dogs. Controlled trials demonstrate that supplementation with *Bacillus subtilis* or *Bacillus coagulans* can modulate fecal microbiota, support stool quality, and maintain gut homeostasis under stressors such as abrupt dietary transition [3,4]. In addition, *Bacillus velezensis* strains have been directly investigated in canine clinical settings. A randomized controlled trial showed that dogs with acute diarrhea recovered faster and exhibited improved dysbiosis indices when fed a diet containing *B. velezensis* DSM 15544 [5]. Another field study demonstrated that a fiber plus *B. velezensis* supplement reduced recurrence of anal sac impaction and associated clinical signs [6]. Symbiotic interventions pairing fermentable fibers with *B. velezensis* have also been shown to improve immune markers and support a favorable microbiota in Beagles under controlled feeding conditions [7]. Together, these studies suggest that *Bacillus*-based probiotics may influence canine GI health predominantly through immunologic and epithelial mechanisms rather than direct antimicrobial effects. 

Cross-species studies provide mechanistic insight into *Bacillus velezensis* activity. In poultry, a multi-strain *B. velezensis* consortium functions as an approved gut flora stabilizer and supports microbial balance [8]. In swine models, *B. velezensis*-derived cell-free supernatants enhance epithelial barrier integrity and modulate immune signaling, including increased tight-junction and mucin-associated markers and reduced pro-inflammatory responses [9,10]. Together, these studies support a conserved, metabolite-driven mechanism involving barrier reinforcement, immune modulation, and epithelial resilience. In dogs, however, gastrointestinal barrier integrity exhibits distinct physiological features that warrant species-specific consideration [11,12,13]. For example, canine inflammatory gastrointestinal conditions are characterized by the disruption of epithelial tight-junction organization, increased paracellular permeability, and dysregulated cytokine signaling, frequently driven by endotoxin exposure and innate immune activation [14,15]. Studies using canine intestinal organoids and gut-on-a-chip systems demonstrate that pro-inflammatory stimuli suppress junctional proteins such as ZO-1 and E-cadherin, impair epithelial renewal programs, and promote barrier leakiness, thereby amplifying mucosal immune activation [14,15]. At the same time, canine epithelial cells retain the capacity to restore barrier function through metabolically supported mechanisms that favor tight-junction assembly, redox balance, and controlled innate signaling, as discussed in canine-focused probiotic and postbiotic evaluations [11,12,16]. These observations underscore the importance of interrogating host-directed probiotic mechanisms directly within canine-relevant epithelial systems.

Although canine and swine GI physiology differ in scale and ecological context, the key regulatory processes—tight-junction assembly, mucin biology, cytokine balance, and controlled innate immune activation—are shared across mammalian species. The existing canine clinical trials with *B. velezensis* therefore support the relevance of translating these mechanistic insights into canine models [5,6,7]. Despite this accumulating evidence base, the functional effects of *B. velezensis* metabolites on canine proximal gastrointestinal epithelium and immune cells remain poorly defined. To specifically interrogate host-directed mechanisms independent of bacterial colonization or physical cell–cell contact, the present study employed cell-free supernatant (CFS) derived from a three-strain *Bacillus velezensis* consortium. This approach enables the isolation of probiotic-derived metabolites—often referred to as postbiotic factors—allowing for the direct assessment of their effects on epithelial barrier function, immune signaling, and cellular renewal. Importantly, prior mechanistic studies have demonstrated that *B. velezensis*-derived CFS modulates tight-junction expression, epithelial repair, and cytokine responses in porcine epithelial and immune cell systems, indicating that secreted metabolites represent a central effector arm of *Bacillus* probiotic activity rather than a requirement for live bacterial contact [9,10]. We hypothesized that CFS derived from three *B. velezensis* strains would strengthen epithelial barrier integrity and promote immunoregulatory responses in canine proximal gastrointestinal epithelium (MCA-B1) and macrophage-like (DH82) cells. Phagocytosis and LPS stimulation assays were performed in DH82 cells, a well-established canine macrophage-like cell line with preserved innate immune and phagocytic functions, whereas MCA-B1 epithelial cells were used exclusively for barrier integrity analyses due to their epithelial origin and validated application in permeability and tight-junction studies [17]. Our objective was to delineate these cellular mechanisms directly in canine systems and thereby clarify how *B. velezensis* metabolites may contribute to maintaining GI immune homeostasis in dogs.

## 2. Results

### 2.1. Enhanced Tight-Junction Protein Levels in MCA-B1 Cells After Exposure to Bacillus Cell-Free Supernatants

**Figure 1:**
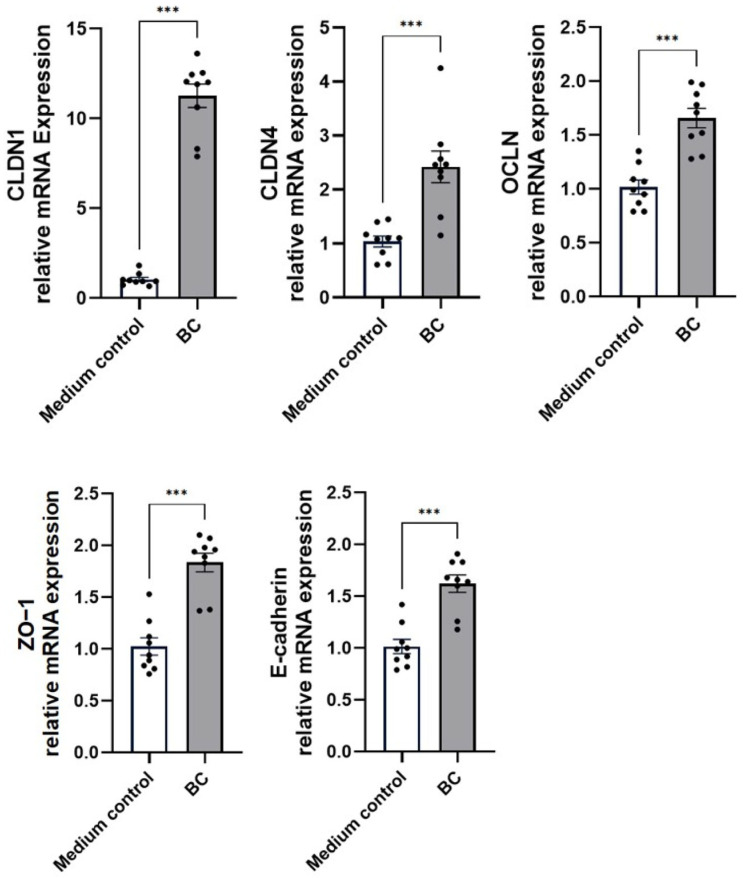
Effect of cell-free supernatant from *Bacillus velezensis* probiotic consortia (BC) on barrier integrity in MCA-B1 cells, assessed by tight-junction mRNA expression (CLDN-1, CLDN-4, OCLN, ZO-1 and E-cadherin), measured by RT-qPCR. Values represent fold change relative to the medium control with standard error (SE) bars. The experiment was performed with 8 replicates per treatment group. *** *p* < 0.001.

The fold-change in mRNA levels of tight-junction (TJ) genes in MCA-B1 cells exposed to cell-free supernatant (CFS) from the *Bacillus* consortia (BC), relative to the medium-only control, is presented in Figure 1. Treatment with BC markedly upregulated several TJ-associated transcripts, including CLDN1 (11.3 ± 0.7-fold; *p* < 0.001), CLDN4 (2.4 ± 0.3-fold; *p* < 0.001), Occludin (1.7 ± 0.1-fold; *p* < 0.001), ZO-1 (1.8 ± 0.1-fold; *p* < 0.001), and E-cadherin (1.6 ± 0.1-fold; *p* < 0.001).

**Figure 2:**
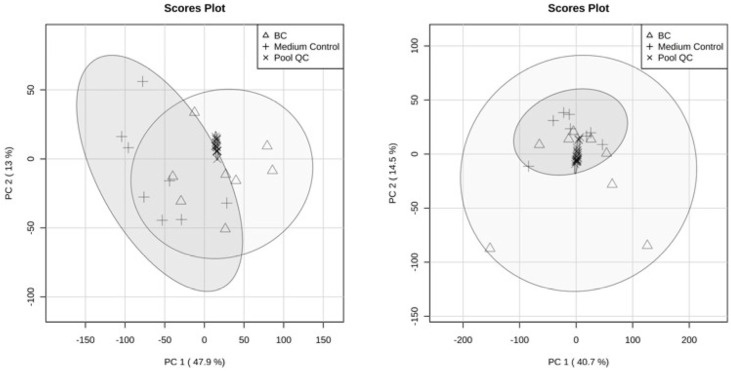
Principal component analysis (PCA) score plots of the analyzed samples.

Label-free quantitative proteome analysis of the two sample series was able to quantify a total of 3909 proteins (without LPS) and 4137 proteins (with LPS). Each dataset was analyzed using multivariate data analysis to evaluate the consistency of the data. Both sets were analyzed in a randomized order and with a pooled sample as QC (pool QC) between samples. The grouping of the pool QC samples aligns near the center of the two plots, indicating that technical variation in the proteomic analysis is smaller than sample variability. In the first PCA model (Figure 2, left panel), the first principal component (PC1) accounted for 47.9% of the total variance in the dataset, while the second principal component (PC2) explained an additional 13.0%. Separation between the BC and medium control groups was primarily observed along PC1, although partial overlap was present. BC samples exhibited greater dispersion across both principal components, indicating increased variability in the proteome profiles relative to the medium control samples. In the second PCA model (right panel), PC1 and PC2 explained 40.7% and 14.5% of the total variance, respectively. Compared with left panel, a greater overlap between the two biological groups was observed, suggesting that the underlying protein abundance patterns show less distinction between condition when cells were stimulated by LPS.

Scores plots showing the distribution of samples along the first two principal components (PC1 and PC2). The left panel (without LPS) displays PC1 (47.9% of explained variance) versus PC2 (13.0%), while the right panel (with LPS) displays PC1 (40.7%) versus PC2 (14.5%). Samples are marked with symbol-coded by group: BC (triangles), medium control (plus symbols), and pool QC (crosses). Shaded ellipses represent the 95% confidence regions for each group, illustrating the within-group variance and overall clustering patterns. The pool QC samples cluster tightly near the center of the score space in both plots, indicating analytical consistency, while BC and medium control samples show broader dispersion and partial separation primarily along PC1.

**Figure 3:**
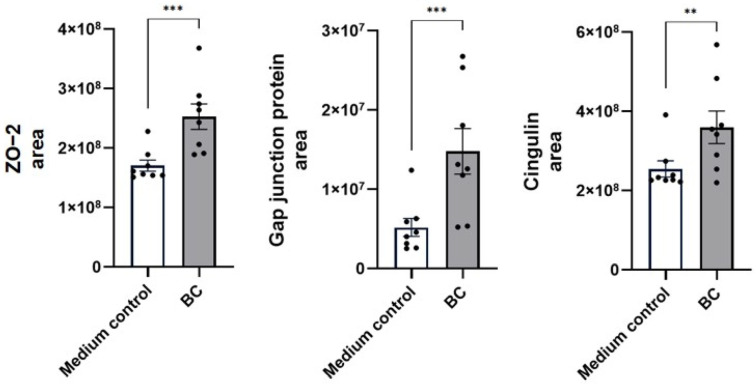
Proteomic abundance of tight-junction protein (ZO-2), the gap junction protein and cingulin in unstimulated MCA-B1 cells after treatment with *Bacillus velezensis* probiotic consortia (BC). Values represent means with standard error (SE) bars. The experiment was performed with 8 replicates per treatment group. ** *p* < 0.01; *** *p* < 0.001.

Figure 3 summarizes the proteomic profile of structural proteins involved in proximal gastrointestinal epithelium cohesion and signaling in MCA-B1 cells following treatment with BC derived CFS (BC-CFS). In the absence of LPS stimulation, BC exposure resulted in elevated levels of ZO-2 (area of treatment means: BC 2.5 ± 0.2 × 10^8^ vs. 1.7 ± 0.1 × 10^8^ in the medium control; *p* < 0.001), gap junction protein (BC 1.5 ± 0.3 × 10^7^ vs. 0.5 ± 0.1 × 10^7^ in the medium control; *p* < 0.001), and cingulin (BC 3.6 ± 0.4 × 10^8^ vs. 2.6 ± 0.2 × 10^8^ in the medium control; *p* < 0.01) compared with the medium control.

**Figure 4:**
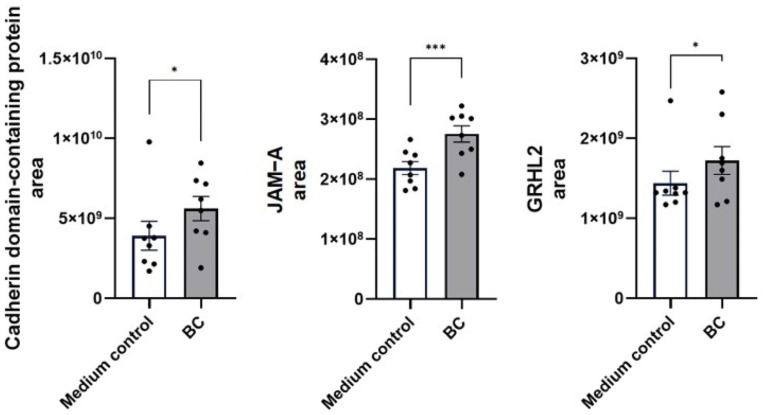
Protein abundance of barrier integrity proteins (cadherin domain-containing protein, JAM-A and GRHL2) in LPS-stimulated MCA-B1 after treatment with *Bacillus velezensis* probiotic consortia (BC). Values represent means with standard error (SE) bars. The experiment was performed with 8 replicates per treatment group. * *p* < 0.05; *** *p* < 0.001.

Under LPS-challenged conditions (Figure 4), BC-CFS further enhanced the abundance of several proteins associated with proximal epithelial barrier function, including cadherin domain-containing protein (BC 5.6 ± 0.8 × 10^9^ vs. 3.9 ± 0.9 × 10^9^ in the medium control; *p* < 0.01), junctional adhesion molecule A (BC 2.8 ± 0.1 × 10^8^ vs. 2.2 ± 0.1 × 10^8^ in the medium control; *p* < 0.001) and grainyhead-like transcription factor 2 ((GRHIL2, BC 1.7 ± 0.2 × 10^9^ vs. 1.4 ± 0.2 × 10^9^ in the medium control; *p* < 0.01).

**Figure 5:**
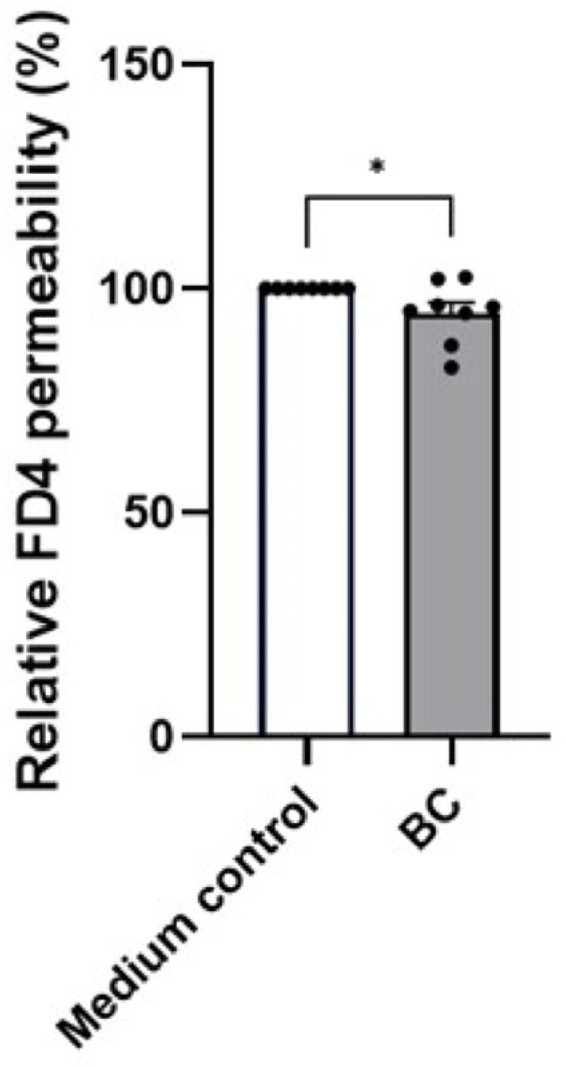
Permeability changes in LPS stimulated MCA-B1 cells treated with *Bacillus velezenis* probiotic consortia (BC). The reduction in permeability (%) was calculated relative to the medium control. The experiment was performed 4 times with a total of 8 replicates per treatment group. Values represent means with standard error (SE) bars. * *p* < 0.05.

To determine whether this BC-mediated elevation of tight-junction components translates into improved proximal intestinal barrier integrity during LPS exposure, a permeability assay was conducted (Figure 5). Pre-incubation of MCA-B1 cells with BC-reduced FITC-Dextran translocation to 94.5 ± 2.4%, compared with the untreated LPS-stimulated cells (set at 100%; *p* < 0.05), indicating partially mitigating LPS-induced permeability.

### 2.2. Induction of Cell-Mediated Immune Cytokines in MCA-B1 Cells by Bacillus velezensis-Derived Metabolites

**Figure 6:**
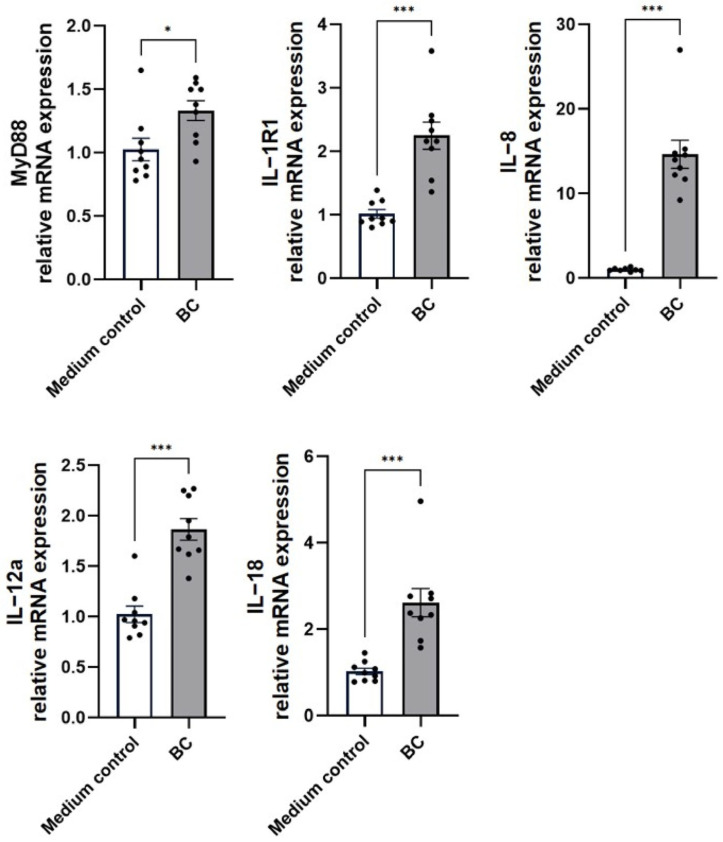
Effect of cell free supernatant from *Bacillus velezenis* probiotic consortia (BC) on the mRNA expression of immunomodulatory cytokines (MyD88, IL-1R1, IL-8, IL-12a and IL-18) in unstimulated MCA-B1 cells, measured by RT-qPCR. Data are expressed as fold change relative to the medium control with standard error (SE) bars. The experiment was performed with 8 replicates per treatment group. * *p* < 0.05; *** *p* < 0.001.

The impact of BC pre-exposure on cytokine transcriptional responses in MCA-B1 proximal intestinal epithelium is presented in Figure 6. Relative to the medium control, cells treated with BC-CFS exhibited significant upregulation of key immune mediators. Specifically, MyD88 expression increased by 33.2% ± 7.8 (*p* < 0.05), while IL-1R1 was elevated 2.2 ± 0.2-fold (*p* < 0.001). Marked induction was also observed for IL-8 (14.6 ± 1.7-fold, *p* < 0.001), IL-12α (1.9 ± 0.1-fold, *p* < 0.001), and IL-18 (2.6 ± 0.3-fold, *p* < 0.001).

**Figure 7:**
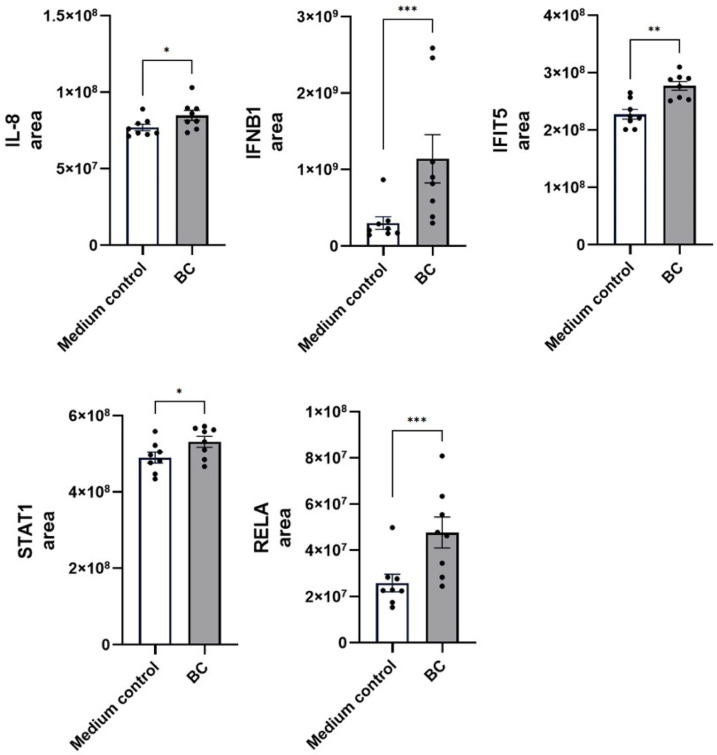
Proteomic abundance of immunostimulatory cytokines and receptor molecules (IL-8, IFNB1, IFIT5, STAT1 and RELA) in unstimulated MCA-B1 cells after treatment with cell-free supernatant from probiotic consortia. Values are means with associated standard error (SE) bars. The experiment was performed with 8 replicates per treatment. * *p* < 0.05; ** *p* < 0.01; *** *p* < 0.001.

Proteomic analyses further corroborate these transcriptional alterations (Figure 7). When compared with the medium control, BC-CFS treatment significantly increased the abundance of IL-8 (BC 8.5 ± 0.3 × 10^7^ vs. 7.7 ± 0.2 × 10^7^ in medium control; *p* < 0.05) and profoundly elevated interferon-β1 (IFNB1) levels (1.1 ± 0.3 × 10^9^ vs. 0.3 ± 0.1 × 10^9^; *p* < 0.001). A similar trend was observed for IFIT5, whose abundance rose from 2.3 ± 0.8 × 10^8^ in controls to 2.8 ± 0.8 × 10^8^ (*p* < 0.01). Moreover, key transcriptional regulators associated with innate immunity and interferon signaling, including STAT1 (5.3 ± 0.1 × 10^8^ vs. 4.9 ± 0.1 × 10^8^; *p* < 0.05) and NF-κB p65 (RelA) (4.8 ± 0.7 × 10^7^ vs. 2.6 ± 0.4 × 10^7^; *p* < 0.001), were also significantly upregulated, reflecting a robust activation of canonical innate immune signaling pathways.

**Figure 8:**
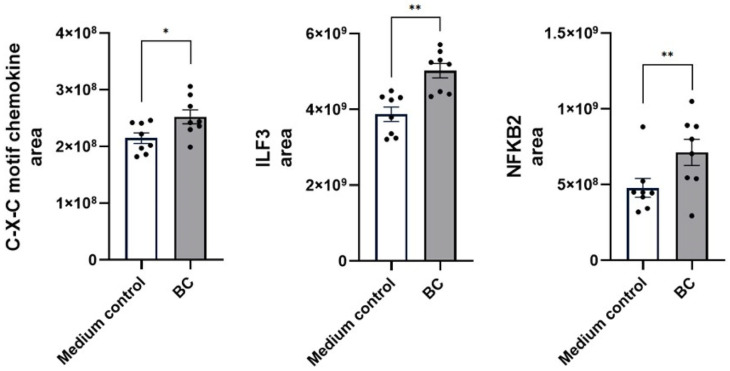
Protein abundance of immune response genes (C-X-C motif chemokine, ILF3 and NFKB2) in LPS-stimulated MCA-B1 cells after treatment with cell free supernatant from probiotic consortia. Values are means with associated standard error (SE) bars. The experiment was performed with 8 replicates per treatment. * *p* < 0.05; ** *p* < 0.01.

In LPS-challenged MCA-B1 cells (Figure 8), supplementation with BC-CFS further amplified the immune response. A substantial increase in C-X-C motif chemokine abundance was observed relative to the untreated, LPS-stimulated medium control (2.5 ± 0.1 × 10^8^ vs. 2.1 ± 0.1 × 10^8^ in medium control; *p* < 0.05). Additionally, BC-CFS significantly elevated the levels of Interleukin Enhancer-Binding Factor 3 (ILF3), rising from 3.98 ± 0.74 × 10^9^ in the medium control to 6.92 ± 1.09 × 10^9^ (*p* < 0.01). A similar response pattern was observed for NF-κB2 (*p* < 0.01).

### 2.3. Enhanced Phagocytic Activity of DH82 Cells in Response to Bacillus velezensis Metabolites

**Figure 9:**
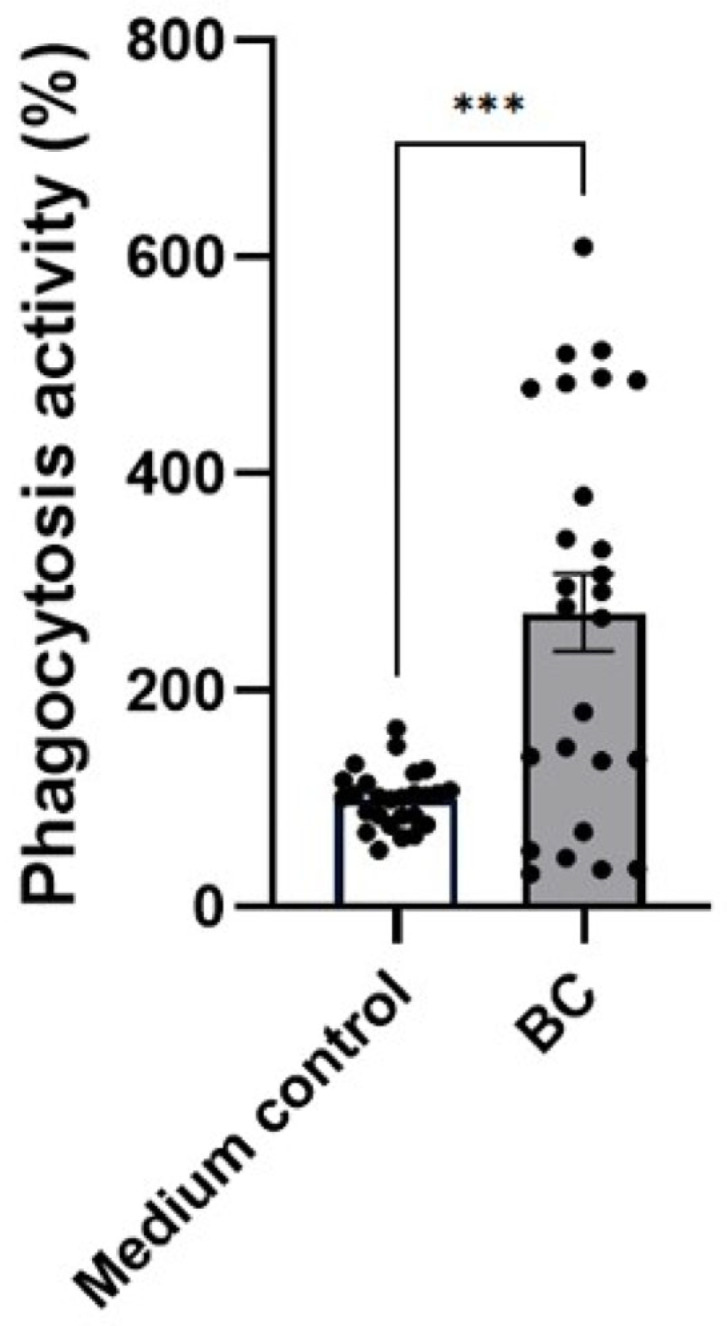
Phagocytic activity percentage of LPS-stimulated DH82 macrophages after treatment with CFS from *Bacillus velezenis* probiotic consortia (BC). Values are representative of mean with associated standard error (SE). The experiment was performed 3 times with 31 replicates in total per treatment group. *** *p* < 0.001.

In unstimulated DH-82 macrophage-like cells, exposure to BC-CFS did not alter basal phagocytic activity. In contrast, when the cells were challenged with LPS (Figure 9), pre-treatment with BC-CFS produced a pronounced enhancement in phagocytic function. Specifically, BC-CFS increased the proportion of phagocytic cells to 271.9 ± 35.6%, compared with the 100 ± 5.1% observed in the LPS-treated medium control (*p* < 0.001).

### 2.4. Anti-Inflammatory Cytokine Induction by Bacillus velezensis-Derived Metabolites in MCA-B1 Cells and LPS-Stimulated DH82 Cells

**Figure 10:**
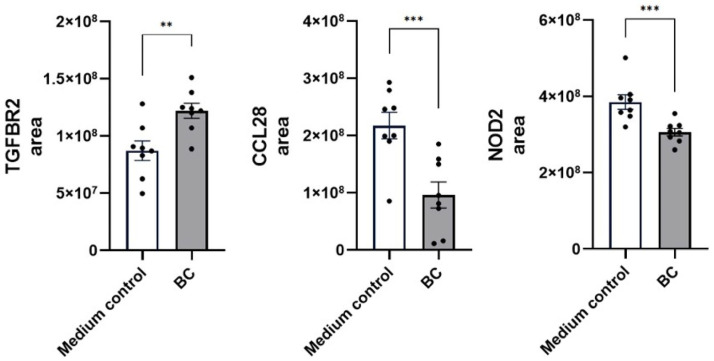
Proteomic abundance of immune regulatory genes (TGFBR2, CCL28 and NOD2) in unstimulated MCA-B1 cells treated with CFS from *Bacillus velezenis* probiotic consortia (BC). Values are means with associated standard error (SE) bars. The experiment was performed with 8 replicates per treatment. ** *p* < 0.01; *** *p* < 0.001.

The ability of *Bacillus velezensis*-derived metabolites to modulate anti-inflammatory cytokine signaling in MCA-B1 cells is shown in Figure 10. Treatment with BC-CFS altered the abundance of several immune-regulatory proteins, including Transforming Growth Factor Beta Regulator 2 (TGFBR2), chemokines, and components of the NOD2 pathway. Compared with the medium control, BC-CFS significantly increased TGFBR2 levels (8.5 ± 0.3 × 10^7^ vs. 7.7 ± 0.2 × 10^7^; *p* < 0.01), while CCL28 abundance was markedly reduced (1.0 ± 0.2 × 10^8^ vs. 2.2 ± 0.2 × 10^8^; *p* < 0.001). A similar reduction was observed for NOD2 (3.1 ± 0.1 × 10^8^ vs. 3.9 ± 0.2 × 10^7^; *p* < 0.001).

**Figure 11:**
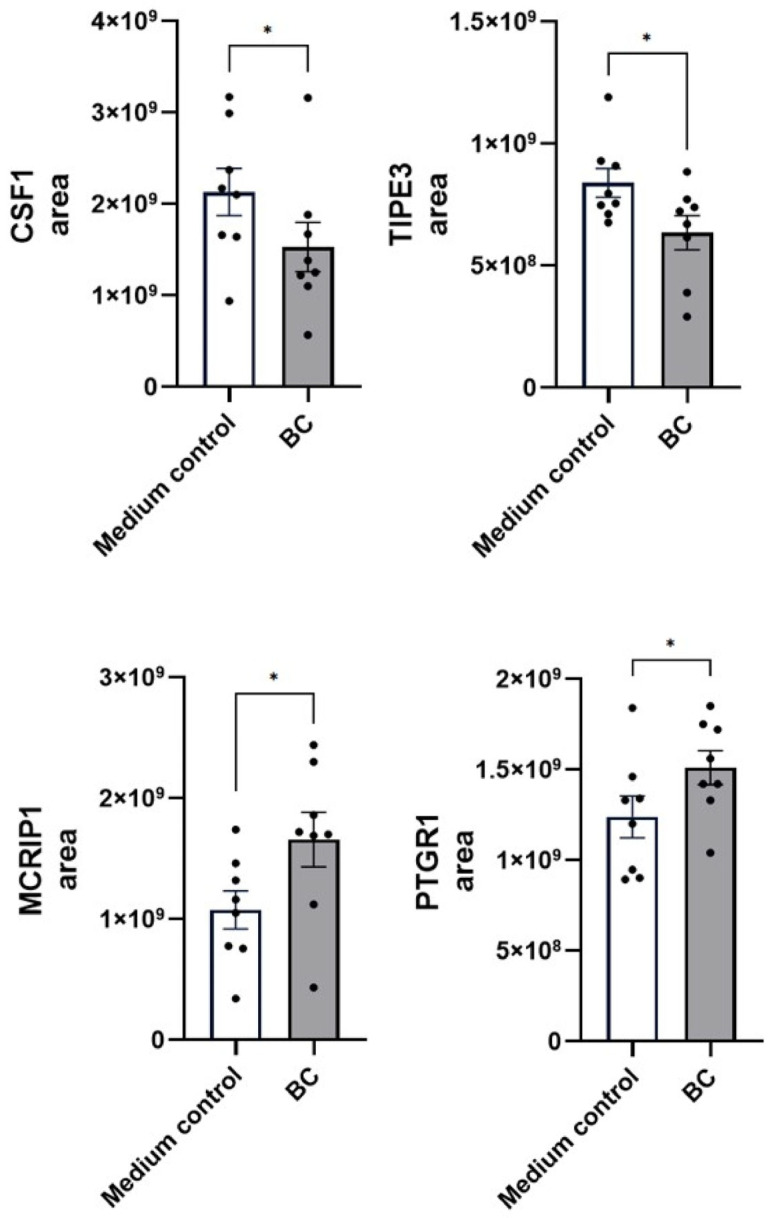
Proteomic abundance of immune regulatory markers (CSF1, TIPE3, MCRIP1 and PTGR1) in LPS-stimulated MCA-B1 cells treated with CFS from *Bacillus velezenis* probiotic consortia (BC). Values are means with associated standard error (SE) bars. The experiment was performed with 8 replicates per treatment. * *p* < 0.05.

**Figure 12:**
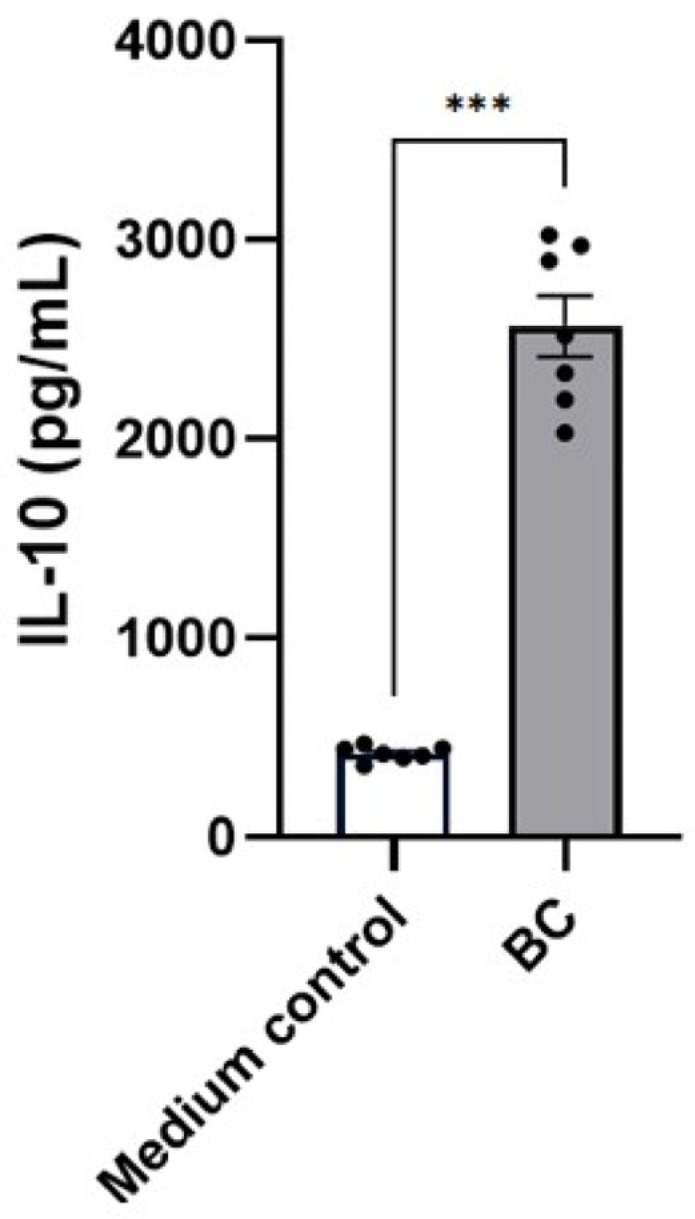
Secreted IL-10 concentration (pg/mL) in LPS-stimulated DH82 macrophages treated with CFS from *Bacillus velezenis* probiotic consortia. The experiment was performed with 7 replicates per treatment. *** *p* < 0.001.

A comparable regulatory pattern emerged in LPS-stimulated MCA-B1 cells (Figure 11), where BC-CFS diminished the abundance of pro-inflammatory macrophage colony-stimulating factor 1 (CSF1, *p* < 0.05) and TNF-alpha-induced protein 8-like 3 (TIPE3, *p* < 0.05). In contrast, BC-CFS enhanced levels of several anti-inflammatory mediators, including MAPK-regulated corepressor-interacting protein 1 (MCRIP1, *p* < 0.05) and Prostaglandin reductase 1 (PTGR1, *p* < 0.05). Furthermore, BC-CFS robustly increased secretion of the anti-inflammatory cytokine IL-10 in LPS-stimulated DH82 cells, reaching 2568 ± 151.2 pg/mL compared with 422.7 ± 13.7 pg/mL in the medium control (*p* < 0.001, Figure 12).

### 2.5. Cell-Free Supernatant from Probiotic Bacillus velezensis Promotes Gene Expression for Proximal Intestinal Epithelial Renewal and Wound Healing in MCA-B1 Cells

**Figure 13:**
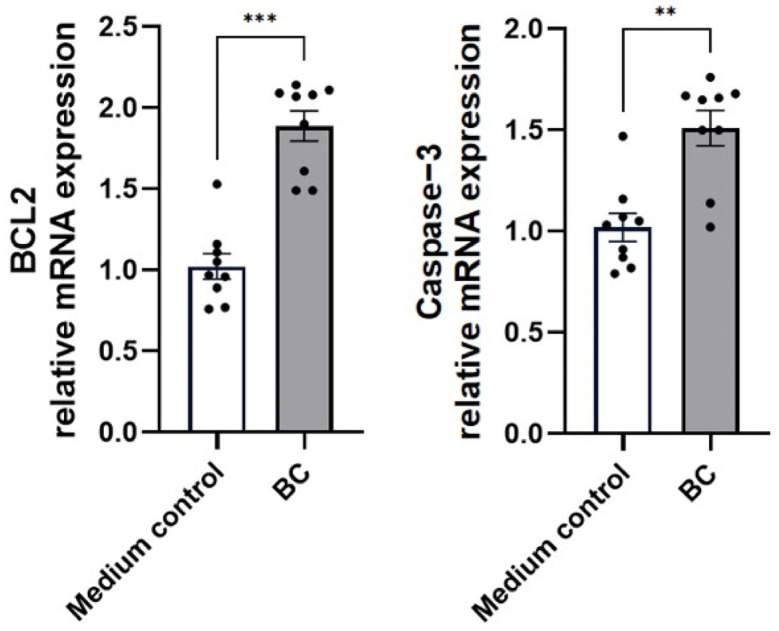

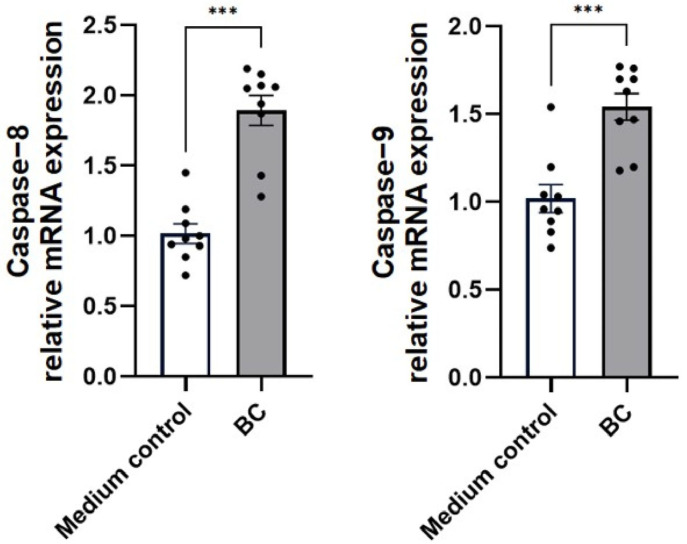
Effect of cell-free supernatant from *Bacillus velezenis* probiotic consortia (BC) on the mRNA expression of anti-apoptotic gene (Bcl-2) and pro-apoptotic genes (caspase-3, caspase-8, caspase-9) in unstimulated MCA-B1 cells, measured by RT-qPCR. Data are expressed as fold change relative to the medium control with standard error (SE) bars. The experiment was performed with 8 replicates per treatment group. ** *p* < 0.01; *** *p* < 0.001.

**Figure 14:**
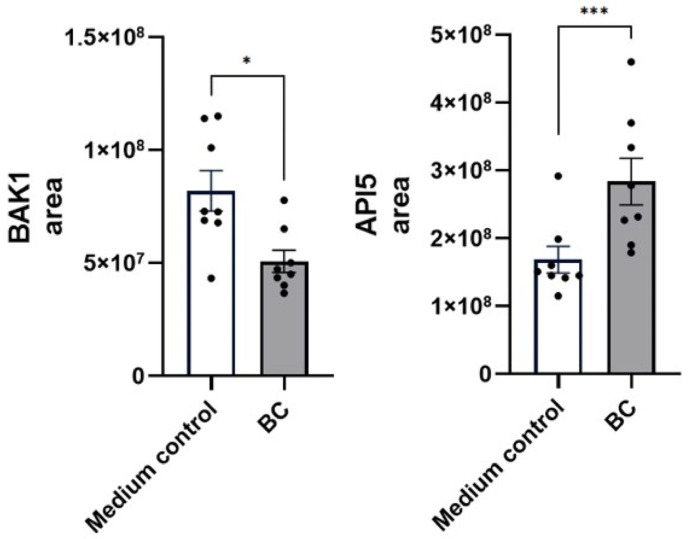

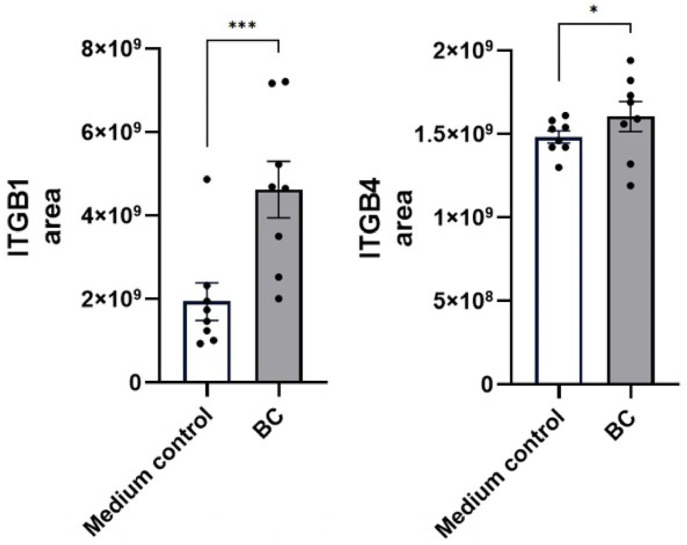
Protein abundance of apoptotic markers (BAK1 and API5) and wound-healing-associated integrins (ITGB1 and ITGB4) in unstimulated MCA-B1 cells treated with CFS from *Bacillus velezenis* probiotic consortia (BC). Values are means with associated standard error (SE) bars. The experiment was performed with 8 replicates per treatment. * *p* < 0.05; *** *p* < 0.001.

The influence of BC-derived CFS on the transcription of apoptosis- and wound-healing-related genes in MCA-B1 cells is presented in Figure 13 and Figure 14. Compared with the medium control, BC-CFS exposure produced a marked elevation in BCL2 mRNA expression (1.9 ± 0.1-fold; *p* < 0.001; Figure 13). In parallel, the pro-apoptotic mediators Caspase-3, Caspase-8, and Caspase-9 were also upregulated, showing fold-increases of 1.5, 1.9, and 1.5, respectively (*p* < 0.01, *p* < 0.001, and *p* < 0.001).

Complementing these transcriptional changes, proteomic quantification confirmed enhanced pro-apoptotic signaling, reflected by a reduction in the abundance of the BCL-2 antagonist BAK1 (5.1 ± 0.5 × 10^7^ vs. 8.2 ± 0.7 × 10^7^ in controls; *p* < 0.05, Figure 14), together with increased levels of the anti-apoptotic regulator apoptosis inhibitor 5 (API5, 1.7 ± 0.2 × 10^8^ vs. 2.8 ± 0.3 × 10^8^ in the medium control; *p* < 0.001). Additionally, proteins associated with proximal intestinal epithelium repair, specifically integrin subunit beta-1 (ITGB1) and integrin subunit beta-4 (ITGB4), were also significantly elevated (*p* < 0.001 and *p* < 0.05, respectively).

**Figure 15:**
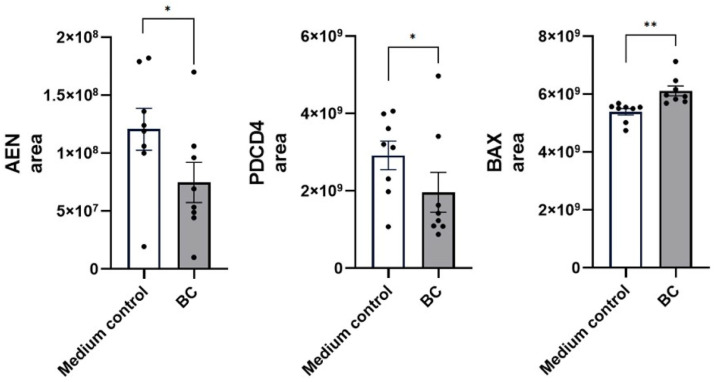

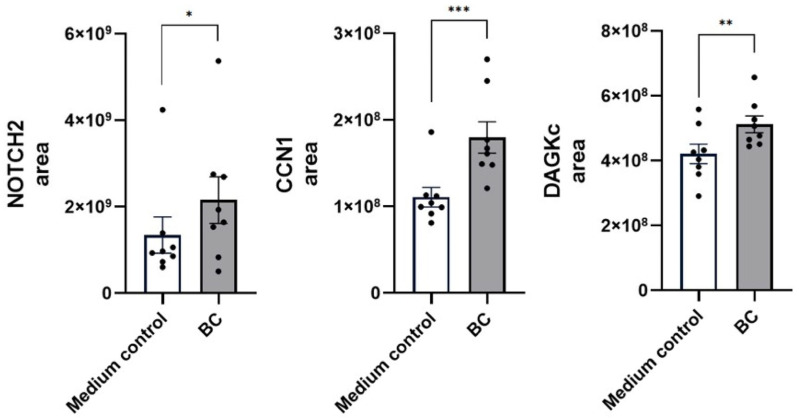
Protein abundance of apoptotic markers (AEN, PDCD4) and wound-healing-associated genes (NOTCH2, CCN1 and DAGKc) in LPS treated MCA-B1 cells. Data are expressed as fold change relative to the medium control with standard error (SE) bars. The experiment was performed with 8 replicates per treatment group. * *p* < 0.05; ** *p* < 0.01; *** *p* < 0.001.

A comparable response pattern was observed in LPS-stimulated MCA-B1 cells (Figure 15). Here, BC-CFS treatment further decreased abundance of apoptosis enhancing nuclease (AEN, 7.5 ± 1.7 × 10^7^ vs. 1.2 ± 0.2 × 10^8^ in controls *p* < 0.05) and programmed cell death protein 4 (PDCD 4, 2.0 ± 0.5 × 10^9^ vs. 2.9 ± 0.4 × 10^9^ in controls, *p* < 0.05). In contrast, the apoptotic marker BAX (6.1 ± 0.2× 10^9^ vs. 5.4 ± 0.1 × 10^9^ in controls, *p* < 0.01) was simultaneously elevated, suggesting an additional layer of apoptotic pathway modulation. With respect to wound-healing responses, BC-CFS enhanced the expression of the regenerative mediators Notch2, CCN1 (CCN family member 1) and DAGKc domain-containing protein, with highly significant increases (*p* < 0.05, *p* < 0.001 and *p* < 0.01, respectively).

### 2.6. Metabolomics Analysis in Bacillus velezensis and MCA-B1 Cell Coculture

**Figure 16:**
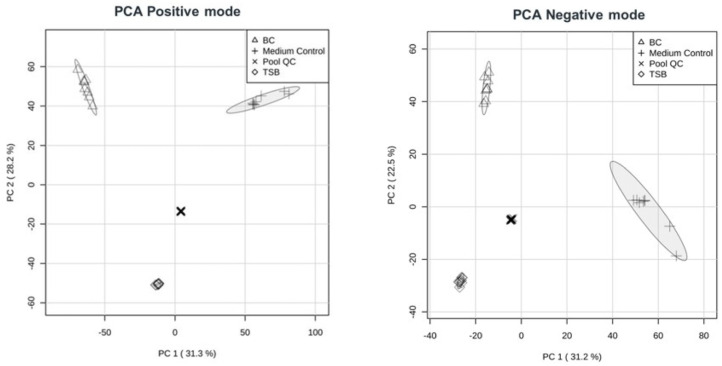
Principal component analysis (PCA) of untargeted metabolomics in positive and negative ionization mode. PCA score plots based on data acquired in positive (**left panel**) and negative ionization modes (**right panel**) show separation between BC and medium control samples.

Untargeted metabolomics was used to globally assess metabolic differences and enable the identification of key metabolites differentiating the experimental groups. Principal component analysis (PCA) was performed separately on metabolomics data acquired in positive and negative ionization modes to explore global metabolic variation among the sample groups (Figure 16). In positive ionization mode (left panel), PC1 and PC2 explained 31.3% and 28.2% of the total variance, respectively, while in negative ionization mode (right panel) PC1 accounted for 31.2% and PC2 for 22.5% of the variance. In both ionization modes, BC samples were clearly separated from the medium control, primarily along PC1, indicating consistent differences in overall metabolic profiles. Samples corresponding to TSB, representing the culture medium, formed a distinct cluster separated mainly along PC2 in both datasets. Tight clustering of replicates and central positioning of the pooled QC samples in both ionization modes support good analytical reproducibility and data stability.

**Figure 17:**
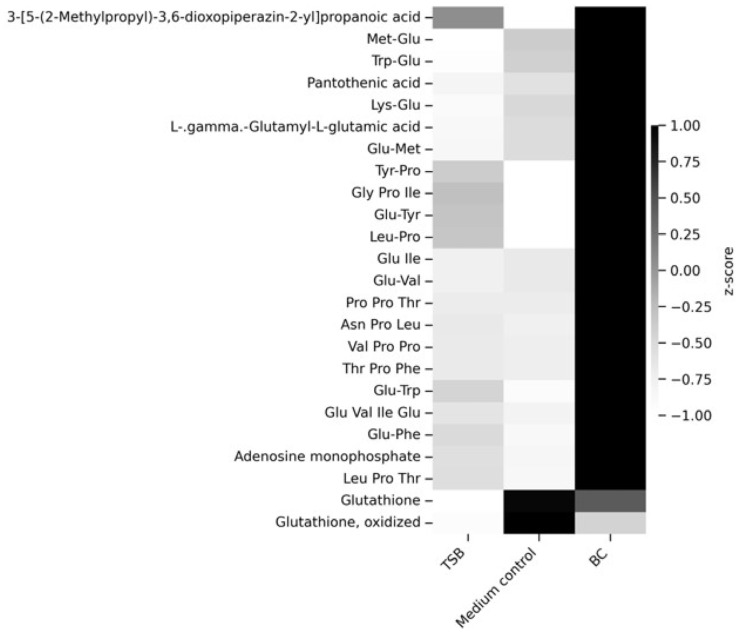
Heatmap visualization of selected metabolites across sample groups. Heatmap displaying z-score-normalized relative abundances of statistically significant metabolites detected in BC, medium control, and TSB samples. Metabolites are shown in rows and sample groups in columns. Color intensity represents relative abundance, with darker shades indicating higher values and lighter shades indicating lower values.

A heatmap based on z-score-normalized metabolite intensities was used to visualize relative abundance patterns across sample groups (Figure 17). Distinct clustering patterns were observed between BC and medium control samples for several metabolites, indicating consistent differences at the metabolite level. The TSB samples showed a separate profile, consistent with their role as culture medium and their distinct compositional background. These patterns align with the PCA and offer additional metabolite-specific information about the dataset’s variability.

The heatmap was generated using annotated metabolites that were identified as significantly different between groups based on univariate statistical analysis using *p*-value (FDR) criteria. This targeted visualization highlights metabolite-level differences underlying the multivariate separation observed in the PCA. Among the significantly altered metabolites, reduced and oxidized glutathione (GSH and GSSG) displayed distinct abundance patterns between BC and medium control samples, suggesting differences in redox balance and oxidative stress-related processes. In addition, pantothenic acid, a precursor of coenzyme A and a key metabolite in central energy metabolism, showed differential abundance between groups, indicating potential alterations in metabolic capacity associated with the BC condition. Several amino acids and short peptides also exhibited coordinated changes, pointing to systematic variation in amino acid and peptide metabolisms. In contrast, the metabolite profile of TSB samples was clearly distinct, consistent with background contributions from the culture medium rather than biological metabolism (Supplementary Table S1).

## 3. Discussion

A key rationale for the present study was the use of *Bacillus velezensis* cell-free supernatant (CFS) rather than live bacteria. While probiotic efficacy in vivo depends on oral delivery of viable organisms, multiple studies demonstrate that many host-beneficial effects of *Bacillus* spp. are mediated through secreted metabolites rather than stable colonization or direct epithelial contact [11,12]. Accordingly, CFS provides a controlled framework to examine metabolite-driven host responses while minimizing confounding effects associated with bacterial proliferation or adherence. This approach is particularly relevant in canine gastrointestinal systems, where spore-forming *Bacillus* species are transient residents and are thought to exert functional effects primarily via metabolic interaction with host tissues [11,18,19]. By focusing on CFS, the present study captures the bioactive molecular output of the *B. velezensis* consortium, enabling direct linkage between defined metabolic signatures and observed improvements in epithelial barrier integrity and immune regulation.

The present data indicate that metabolites present in the *Bacillus* consortia (BC) CFS elicit a coordinated epithelial response consistent with enhanced barrier competence in a canine-relevant system. Transcriptional upregulation of canonical tight-junction (TJ) genes (CLDN1, CLDN4, OCLN, ZO-1, E-cadherin) together with increased abundance of junctional/adhesion proteins (ZO-2, cingulin, gap-junction protein, JAM-A) and reduced LPS-induced paracellular flux supports a primary barrier-stabilizing action of BC metabolites. These outcomes align with contemporary canine epithelial platforms, where pro-inflammatory cytokines compromise stemness, depress ZO-1/E-cadherin, and increase permeability in organoids, and where gut-on-chip systems retaining robust junctional architecture under flow better withstand microbial challenge [14,15].

Mechanistically, the marked increase in adenosine monophosphate (AMP) observed in the metabolome provides a plausible upstream cue for the structural phenotype. In intestinal epithelium, AMP-activated protein kinase (AMPK) accelerates TJ reassembly, improves transepithelial electrical resistance, and limits paracellular leakage; thus, an AMP/AMPK axis parsimoniously accounts for the concordant TJ transcript/protein gains and the measured attenuation of LPS-induced permeability. The concomitant elevation of ITGB1/ITGB4 is consistent with strengthened epithelial anchoring and hemidesmosomal stabilization, features linked to re-epithelialization and mechanical cohesion [20,21].

A second driver is nutritive fueling via PEPT1. The BC-conditioned metabolome was enriched in Pro-rich and Glu-linked di-/tripeptides (for example, Leu-Pro, Val-Pro-Pro, Tyr-Pro, Glu-Phe/Glu-Tyr/Glu-Trp/Glu-Ile, Glu-Val-Ile-Glu), consistent with rapid apical uptake and deployment as amino-nitrogen and energetic substrates during restitution—behavior characteristic of PEPT1 and especially relevant under epithelial stress. Several Pro-containing dipeptides also exhibit DPP-IV-modulatory activity in food systems, a property compatible with the balanced inflammatory profile observed here, although not the primary endpoint of the present work [22,23]. Sustained intracellular availability of small peptides supports protein synthesis, cytoskeletal remodeling, and junctional turnover, processes that align closely with the observed increases in claudins, Occludin, ZO-1/ZO-2, JAM-A, and E-cadherin. The simultaneous elevation of multiple peptide species suggests a cumulative metabolic contribution to proximal intestinal epithelial barrier reinforcement rather than a single signaling-driven effect.

BC-CFS further calibrated to proximal intestinal epithelial immunity toward a sentinel yet controlled state. Upregulation of MyD88, IL-1R1, IL-8 and interferon-axis proteins (IFNB1, IFIT5, STAT1) signifies improved pattern recognition and antiviral readiness. This immune activation coincided with metabolomic enrichment of both Pro- and Glu-containing di- and tripeptides, which likely support the biosynthetic and energetic demands associated with cytokine production. In parallel, increased IL-10 and TGFBR2 with reductions in CCL28 and NOD2, together with attenuation of M-CSF1 and TNFAIP8-like 3 during LPS, point to active resolution rather than unchecked activation. Comparable “defend-but-not-damage” equilibria—preserving junctional morphology under microbial inputs—are reported in canine epithelial platforms, underscoring the physiological relevance of the immune set-point achieved here [14,16].

The pronounced enhancement of phagocytic activity in LPS-challenged DH82 macrophage-like cells indicates that BC-derived metabolites may also support immune effector functions. Although direct metabolite uptake by macrophages was not assessed, several enriched metabolites in the BC-conditioned coculture are consistent with immune modulation. In particular, cyclic peptides such as cyclo (gly-pro-glu) and diketopiperazine-related compounds were substantially elevated. Such cyclic peptides have been reported to influence immune cell metabolism, cytoskeletal dynamics, and redox signaling, thereby supporting efficient phagocytosis and pathogen handling [24,25]. These metabolic inputs may therefore contribute to the enhanced phagocytic competence observed following BC-CFS exposure.

The redox signature complements these findings. Elevated γ-glutamyl peptides and pantothenic acid, together with significantly lower GSSG, indicate reinforcement of glutathione-centric buffering (γ-glutamyl cycle) and coenzyme-A-linked lipid renewal—conditions associated with restrained NF-κB/p38 signaling and improved epithelial survival under oxidative/inflammatory stress [26,27,28]. In canine-focused reviews, probiotic interventions are frequently linked to barrier fortification and modulation of host immunity/metabolism, supporting the translational plausibility of a metabolite-driven redox component in barrier protection [11,12].

BC-CFS treatment also promoted proximal intestinal epithelial renewal and wound-healing responses while maintaining controlled apoptosis. This was evidenced by coordinated regulation of BCL2-family proteins and increased abundance of integrins ITGB1 and ITGB4, which are essential for epithelial adhesion and mechanical stability. Among the identified metabolites, pantothenic acid exhibited one of the strongest increases under BC treatment. As a precursor of coenzyme A, pantothenic acid is central to mitochondrial metabolism, lipid synthesis, and membrane remodeling—processes required for epithelial restitution and integrin-mediated anchoring. Enhanced pantothenic acid availability therefore provides a metabolic rationale for the observed reinforcement of epithelial repair pathways without compromising barrier integrity [12].

Finally, the present observations converge with companion work on the same *B. velezensis* consortia. In porcine epithelial–macrophage coculture, Walker and colleagues reported that consortia CFS suppressed enteric pathogens, enhanced TEER, increased TJ protein expression, and modulated cytokine signaling—mechanistic themes coherent with the canine epithelial outcomes reported here and consistent with efficacy mediated by probiotic metabolites at the mucosal interface [10]. In addition, recent canine-focused syntheses and in vitro/in vivo studies emphasize that probiotic strategies can fortify epithelial barrier function, modulate immunity, and remodel microbial/host metabolite profiles, while calling for more mechanism-anchored canine research in advanced organoid-chip systems [15,16].

While the present study demonstrates that *Bacillus velezensis*-derived metabolites directly modulate barrier components, immune signaling, redox balance, and renewal pathways in the canine proximal intestinal epithelium and macrophage-like cells, these findings should be interpreted within the context of an in vitro 2D model. Such systems enable mechanistic dissection of proximal intestinal epithelial and innate immune cell-intrinsic responses but do not capture key features of the gastrointestinal environment, including mucus architecture, mechanical forces, epithelial and immune cell heterogeneity, and dynamic host–microbiome interactions [14,15]. Recent advances in canine intestinal organoid and gut-on-chip platforms highlight the importance of these dimensions for physiological barrier integrity and immune regulation within the intestinal epithelium [16]. Accordingly, the current data provide a mechanistic foundation that complements emerging higher-order canine models and supports future validation in organoid-based systems and in vivo studies, as advocated in contemporary canine probiotic and postbiotic research [11,12].

Taken together, the findings indicate that *Bacillus* consortia-derived metabolites enhance proximal intestinal epithelium resilience through coordinated effects on barrier structure, immune tone, and redox balance. The CFS establishes conditions that support junctional integrity and controlled innate signaling while maintaining oxidative stability during challenge. Within canine-relevant proximal gastrointestinal epithelial systems, these outcomes provide mechanistic evidence that BC metabolites help preserve mucosal homeostasis. In conjunction with companion studies employing the same *B. velezensis* consortia, the present results support the rationale for pursuing metabolite-based *Bacillus* strategies as targeted modulators of canine gastrointestinal barrier function, while acknowledging the anatomical scope of the epithelial model used.

## 4. Materials and Methods

### 4.1. Reagents and Materials

Unless otherwise specified, all cell culture media, consumables and reagents were obtained from Thermo Fisher Scientific (Roskilde, Denmark).

### 4.2. Cell Culture

The canine proximal epithelial cell line MCA-B1 (DSMZ, Braunschweig, Germany; ACC 828) was cultured in DMEM/F12 medium supplemented with 10% heat-inactivated fetal bovine serum (FBS) and 1% penicillin–streptomycin (100 U/mL penicillin and 100 µg/mL streptomycin).

The macrophage-like canine cell line DH82 (LGC Standards GmbH, Wesel, Germany; CRL3590) was maintained under the same growth conditions. All cultures were incubated at 37 °C in a humidified incubator with 5% CO_2_.

### 4.3. Probiotic Strains and Preparation of Cell Free Supernatants (CFS)

Three proprietary *Bacillus velezensis* strains—LSSA01, 15AP4 and 2084—forming part of Enviva^®^ Pro (Danisco Animal Nutrition & Health, IFF, Oegstgeest, The Netherlands) were used in this study.

Each strain was grown aerobically overnight at 37 °C in Tryptic Soy Broth (TSB). Optical density at 600 nm (OD_600_) was recorded using an EnSight Multimode Plate Reader (PerkinElmer, Shelton, CT, USA). Cultures were centrifuged, and supernatants were sterilized through 0.2 μm vacuum filtration (Thermo Scientific Nalgene aPES membrane, Thermo Scientific, Waltham, MA, USA).

A probiotic consortium was prepared by mixing the CFS from the three strains in equal proportions. The CFS preparations were stored at −20 °C until further use. CFS was added at ≤5% (*v*/*v*) to complete culture medium; preliminary measurements confirmed that the final coculture pH remained within 7.0–7.2, and therefore no pH adjustment was performed. OD_600_ was calibrated to CFU by serial dilution and agar plate enumeration (OD_600_ = 1.0 ≈ 1 × 10^9^ CFU/mL). CFS was applied to all assays at a working concentration equivalent to 1 × 10^7^ CFU/mL.

### 4.4. RT-qPCR Analysis of Gene Expression in MCA-B1 Cells

MCA-B1 proximal epithelial cells were seeded at 2 × 10^5^ cells/mL in 96-well plates and allowed to reach confluency at 37 °C. On day 2, cells were treated with CFS at a concentration equivalent to the secreted fraction from 1 × 10^7^ CFU/mL, based on OD_600_ calibration. Control wells received DMEM with TSB but no CFS. Plates were incubated overnight in 5% CO_2_. After treatment, cells were washed with PBS and lysed in 200 µL RLT buffer (Qiagen, Kastrup, Denmark) for 30 min at 37 °C; proteinase K (100 µg/mL final concentration) was then added. RNA extraction and RT-qPCR were performed by Biotest (Trige, Denmark) using TaqMan assays listed in Supplementary Table S2, following previously established workflows.

The expression panel included cytokines (IL8, IL12a, IL18), tight-junction genes (CLDN1, CLDN4, OCLN, ZO-1, E-cadherin), apoptosis markers (BCL2, caspase-3, caspase-8, caspase-9) and immunomodulators (MyD88, IL1R1). Each condition was run with eight biological replicates. Gene expression was normalized to three housekeeping genes (Supplementary Table S2) using the following:

ΔCt = Ct_target − Ct_housekeeping. Relative gene expression was calculated as Fold change = 2^−(ΔCt_sample − ΔCt_control)^ using the 2^−ΔΔCt^ method.

### 4.5. Proteomic Analysis of MCA-B1 Cells

MCA B1 cells were seeded as described above and cultured to confluency. Media were refreshed, and CFS was added at a concentration equivalent to 1 × 10^7^ CFU/mL. For stimulated conditions, LPS (50 ng/mL) was added to half of the treatment wells. Plates were incubated overnight at 37 °C, 5% CO_2_. Control wells contained DMEM + TSB only. Each condition included eight replicates.

Cells were washed with PBS and lysed in a buffer containing 5% SDS, 100 mM triethylammonium bicarbonate and protease inhibitors. Proteomic profiling was performed following the workflow of Kadekar et al. [29]. Peptides were analyzed using a Vanquish Neo UHPLC system coupled to a QExactive HF mass spectrometer (Thermo Scientific, Waltham, MA, USA). Samples were separated using a trap and elute configuration with a NanoViper trap column and a Waters nanoEase M/Z Peptide BEH C18 column. A 70 min chromatographic gradient at 2000 nL/min was used. Mass spectrometry was carried out in DDA mode with HCD fragmentation.

Raw data were processed in Proteome Discoverer 3.0 and searched against a UniProt Canis familiaris FASTA database using Mascot (version 3.1). Label-free quantification (LFQ) was performed in Expressionist v.2025.1.3 (Genedata, Basel, Switzerland). Imported raw files were noise-filtered using a chemical noise subtraction. Chromatograms were retention-time aligned by a pairwise alignment, filtered and smoothed before peak detection, based on volumes. Detected peaks were isotopic clustered and singletons were filtered out. Peak clusters were matched to the identifications from Proteome Discoverer. Proteins were quantified based on the three most intense peptides. Quantitative abundances were normalized by an intensity drift normalization, where intensities from QC samples were used to correct for drift in the nano LC-MS/MS analysis.

### 4.6. Permeability Assay

Permeability was assessed using FITCdextran (4 kDa) (Merck Life Science A/S, Søborg, Denmark). MCA-B1 cells were seeded on polyester Transwell inserts (0.33 cm^2^, 0.4 µm pore size; Corning) at 2 × 10^5^ cells/well and cultured to confluency. Cells were then treated with CFS an equivalent concentration to 1 × 10^7^ CFU/mL or left untreated for 24 h. Afterward, monolayers were stimulated with 50 ng/mL LPS or remained unchallenged. The LPS concentration (50 ng/mL) was selected based on prior validation in canine epithelial and macrophage-like cell models, where it elicits robust inflammatory and barrier responses without inducing cytotoxicity [17] and Supplementary Figure S1. After another 24 h, apical medium was replaced with 1 mg/mL FITC-dextran, and basolateral medium was collected after 4 h. Fluorescence was measured using a SpectraMax i3x reader (excitation 485 nm, emission 535 nm, Molecular Devices, CA, USA), and concentrations were interpolated from a standard curve. Permeability was calculated relative to untreated controls:

Permeability (%) = (FD4 _in test sample_/Mean FD4 _in untreated sample_) × 100

The assay was performed in 4 independent experiments with 2 biological replicates per treatment per run (total 8 replicates per condition).

### 4.7. Phagocytosis Assay

Phagocytic activity of DH82 cells was quantified using the Vybrant Phagocytosis Assay Kit (Thermo Fisher, Waltham, MA, USA). Cells were seeded at 2 × 10^5^ cells/mL in 96-well plates and incubated overnight. After media replacement, CFS was added to achieve an equivalent concentration to 1 × 10^7^ CFU/mL; half of the wells also received LPS (50 ng/mL). Cells were incubated for 24 h at 37 °C.

Fluorescein-labeled *E. coli* K12 BioParticles were then added for 2 h, after which extracellular fluorescence was quenched with 0.4% trypan blue. Blank wells (media only) were included to determine background fluorescence. The assay was repeated three times, with 32 total replicates. Fluorescence was measured at 480 nm/520 nm and phagocytic activity was calculated as:

Phagocytosis (%) = [(Fluorescence__test_ − Fluorescence__blank_)/(Fluorescence__untreated_ − Fluorescence__blank_)] × 100

### 4.8. IL-10 Measurement by Multiplex in LPS Stimulated DH-82 Cells

DH-82 cells were seeded at 2 × 10^5^ cells/mL in 96-well plates and allowed to reach confluency at 37 °C. Cells were then treated with CFS at a concentration equivalent to the secreted fraction from 1 × 10^7^ CFU/mL, based on OD_600_ calibration. Control wells received DMEM with TSB but no CFS. LPS (50 ng/mL) was added along in both BC and medium control conditions. Supernatants were collected after treatment, centrifuged at 5000× *g* for 10 min, and stored at −20 °C until analysis.

Canine IL-10 concentrations in culture supernatants were measured using a bead-based multiplex cytokine immunoassay (MILIPLEX, Sigma, Søborg, Denmark). Standards and samples and quality controls were loaded into a 96-well plate with Assay buffer, followed by addition of fluorescent magnetic beads coupled to anti-canine IL-10 antibodies (bead region 63) and incubated overnight at 4 °C. After incubation with shaking, plates were washed using a magnetic manifold and sequentially incubated with biotinylated detection antibodies and streptavidin-phycoerythrin. Following the final wash, beads were resuspended in sheath fluid and analyzed on a Bio-Plex 200-HTF (Bio-Rad, Copenhagen, Denmark), acquiring a minimum of 50 beads per analyte. IL-10 concentrations were calculated using a five-parameter logistic (5-PL) standard curve and reported in pg/mL. Each experimental condition contained 7 biological replicates, analyzed in technical duplicates.

### 4.9. Untargeted Metabolomics of Cell–CFS Coculture Supernatants

MCA-B1 cells were seeded at 2 × 10^5^ cells/mL in 96-well plates and cultured to confluency. Fresh medium was added, followed by CFS (equivalent to 1 × 10^7^ CFU/mL). After 24 h at 37 °C, supernatants were collected, vortexed, and centrifuged at 5000× *g* for 5 min and filtered through a 96-well filter plate with 0.2 μM filter (Sigma, Brøndby, Denmark). Samples were randomized and diluted 1:1 with H_2_O:MeOH using an Opentrons Flex liquid handler (Opentrons Labworks Inc., Long Island City, NY, USA). A pooled QC sample was prepared by combining 10 µL of each sample, and was injected after every ten analytical injections.

Metabolomic analysis was performed with a 1290 Infinity III UHPLC (Agilent, Glostrup, Denmark) coupled to a timsTOF Flex MALDI 2 mass spectrometer (Bruker, Roskilde, Denmark). Separation was achieved on an Acquity UPLC HSS T3 column using a water–acetonitrile gradient containing 0.1% formic acid. Injection volume was 5 µL; flow rate 0.25 mL/min. Mass spectrometry was run in positive and negative modes using a VIP HESI source and MS/MS fragmentation was acquired using Parallel Accumulation–Serial Fragmentation (PASEF, 100–1350 *m*/*z*). Internal calibration was performed using sodium formate and an Agilent low-concentration tuning mix.

Data processing was completed in MetaboScape (v2026) using the T-Rex 4D algorithm (*m*/*z*, retention time, ion mobility and intensity). Feature annotation used a combination of inhouse MS^2^ libraries, GNPS, LipidBlast, Bruker NIST HRMS, MetaboBase Personal Library 3.0 and HMDB.

### 4.10. Statistical Analysis

All datasets were analyzed using the Kruskal–Wallis H test (non-parametric one-way ANOVA on ranks). When multiple comparisons were performed, the false discovery rate (FDR) was controlled at 5%. Statistical analyses were conducted in GraphPad Prism version 9. A *p* value < 0.05 was considered statistically significant. PCA plots were created using MetaboAnalyst 4.0 (www.metaboanalyst.ca; accessed on 7 April 2026). Data was normalized, log-transformed, and auto-scaled.

## Data Availability

The data supporting the findings of this study are included within the article and its Supplementary Materials. Additional datasets generated and/or analyzed during the current study are available from the corresponding authors upon reasonable request.

## Supplementary Materials

The following supporting information can be downloaded at https://www.mdpi.com/article/10.3390/ijms27104417/s1.

## Abbreviations

The following abbreviations are used in this manuscript:

Abbreviation	Full Term
AMP	Adenosine monophosphate
AMPK	AMP-activated protein kinase
API5	Apoptosis inhibitor 5
BAK1	BCL-2 antagonist/killer 1
BAX	BCL-2-associated X protein
BC	*Bacillus velezensis* consortia
BCL2	B-cell lymphoma 2
CFS	Cell-free supernatant
CLDN1/CLDN4	Claudin-1/Claudin-4
DH82	Canine macrophage-like cell line
FITC-Dextran	Fluorescein isothiocyanate–dextran
GSH	Reduced glutathione
GSSG	Oxidized glutathione
IFIT5	Interferon-induced protein with tetratricopeptide repeats 5
IFNB1	Interferon beta-1
IL	Interleukin
IL-10	Interleukin-10
IL-18	Interleukin-18
ITGB1/ITGB4	Integrin subunit beta-1/beta-4
JAM-A	Junctional adhesion molecule A
LPS	Lipopolysaccharide
MCA-B1	Canine epithelial cell line
M-CSF1	Macrophage colony-stimulating factor 1
MyD88	Myeloid differentiation primary response 88
NF-κB	Nuclear factor kappa-B
PEPT1	Proton-coupled oligopeptide transporter 1
PTGR1	Prostaglandin reductase 1
qRT-PCR	Quantitative reverse-transcription PCR
STAT1	Signal transducer and activator of transcription 1
TJ	Tight-Junction
TGFBR2	Transforming growth factor beta receptor 2
TSB	Tryptic soy broth
ZO-1/ZO-2	Zonula occludens-1/Zonula occludens-2
