# Supplementary material for: Functional Effects of Bacillus velezensis Metabolites on Barrier Formation, Cytokine Responses, and Phagocytic Activity in Canine Epithelial and Immune Cells
Source: Int J Mol Sci. 2026 May 15;27(10):4417. doi: 10.3390/ijms27104417 (PMC13207524; doi:10.3390/ijms27104417)
Supplement: Supplementary file 1 [file ijms-27-04417-s001.zip › ijms-4226535-supplementary.pdf]

Supplementary Materials

Table S1. Metabolite abundance detected in BC and MCA-B1coculture.

Metabolites	TSB	Medium control	BC	Fold Change
3-[5-(2-methylpropyl)-3,6-dioxopiperazin-2-yl]propanoic acid	2.5 ± 0.1 x 10^{5}	2.6 ± 1.2 x 10^{4}	4.5 ± 0.2 x 10^{5***}	17.3
Met-Glu	1.4 ± 1.3 x 10^{3}	1.9 ± 0.4 x 10^{4}	6.9 ± 0.7 x 10^{4***}	3.6
Trp-Glu	ND	3.0 ± 0.3 x 10^{3}	1.3 ± 0.1 x 10^{4***}	4.3
Pantothenic acid	2.7 ± 2.0 x 10^{4}	8.6 ± 3.4 x 10^{4}	5.6 ± 0.2 x 10^{5***}	6.5
Lys-Glu	ND	8.7± 1.1 x 10^{2}	4.7 ± 0.5 x 10^{3***}	5.4
γ-glutamyl-L-glutamate	10 ± 0.7 x 10^{3}	1.4 ± 0.1 x 10^{4}	3.3 ± 0.3 x 10^{4***}	2.4
Glu-Met	2.2 ± 0.3 x 10^{4}	3.5 ± 0.4 x 10^{4}	1.1 ± 0.1 x 10^{5***}	3.1
Tyr-Pro	3.7 ± 0.3 x 10^{4}	1.1 ± 0.8 x 10^{3}	14 ± 0.4 x 10^{4***}	127.3
Gly Pro Ile	2.4 ± 0.1 x 10^{5}	ND	7.2 ± 0.3 x 10^{5}	-
Glu-Tyr	8.0 ± 0.3 x 10^{4}	4.3 ± 0.6 x 10^{4}	1.6 ± 0.1 x 10^{5***}	3.7
Leu-Pro	4.8 ± 0.3 x 10^{5}	7.9 ± 3.7 x 10^{3}	1.6 ± 0.1 x 10^{6***}	200
Glu Ile	2.1 ± 0.4 x 10^{4}	3.2 ± 0.6 x 10^{4}	3.2 ± 0.2 x 10^{5***}	10.0
Glu-Val	ND	2.2 ± 1.3 x 10^{3}	6.4 ± 0.4 x 10^{4***}	29.1
Pro Pro Thr	1.8 ± 1.0 x 10^{3}	3.6 ± 3.6 x 10^{2}	4.7 ± 0.5 x 10^{5***}	1305
Asn Pro Leu	2.0 ± 0.6 x 10^{4}	1.4 ± 0.6 x 10^{3}	4.9 ± 0.5 x 10^{5***}	350
Val Pro Pro	5.0 ± 0.5 x 10^{3}	2.3 ± 0.9 x 10^{2}	2.0 ± 0.1 x 10^{5***}	870
Thr Pro Phe	ND	ND	6.7 ± 0.4 x 10^{2}	-
Glu-Trp	1.3 ± 0.1 x 10^{4}	4.8 ± 0.3 x 10^{3}	4.6 ± 0.2 x 10^{4***}	9.5
Glu Val Ille Glu	3.6 ± 1.8 x 10^{4}	9.2 ± 4.8 x 10^{3}	3.2 ± 0.1 x 10^{4***}	3.5
Glu-Phe	1.5 ± 0.1 x 10^{5}	7.0 ± 0.9 x 10^{4}	5.8 ± 0.3 x 10^{5***}	8.3

Adenosine monophosphate	$2.1 \pm 1.4 \times 10^{3}$	$3.7 \pm 2.8 \times 10^{2}$	$1.5 \pm 0.1 \times 10^{4***}$	40.5
Leu Pro Thr	$1.6 \pm 0.4 \times 10^{4}$	$1.3 \pm 1.3 \times 10^{2}$	$1.2 \pm 0.2 \times 10^{5***}$	923
Glutathione. reduced (GSH)	$2.3 \pm 2.3 \times 10^{2}$	$5.8 \pm 0.5 \times 10^{3}$	$4.6 \pm 0.3 \times 10^{3*}$	0.8
Glutathione. oxidized (GSSG)	ND	$6.8 \pm 0.7 \times 10^{5}$	$1.5 \pm 0.1 \times 10^{5***}$	0.2
GSH/GSSG ratio	ND	$9.3 \pm 1.4 \times 10^{-3}$	$31 \pm 1.5 \times 10^{-3***}$	3.3

Values represent mean abundance± SEM. Statistical significance reflects comparison between medium control and BC. TSB values are shown as reference. *, $P < 0.05$; ***, $P < 0.001$; ND, Not detected.

Table S2. Taq Man ID numbers and housekeeping genes used in the RT-qPCR.

Genome target in MCA-B1 cells	TaqMan ID No.
Claudin-1	Cf02713195_u1
Claudin-4	Cf02695489_s1
Occludin	Cf02624089_m1
ZO-1	Cf01552709_m1
E-cadherin	Cf02697525_m1
MyD88	Cf02675965_m1
CCL2	
IL-6	
IL-8	Cf02624262_m1
IL-12	Cf02690011_m1
IL-18	Cf02624262_m1
IL-1R	Cf02647245_m1
Bcl-2	Cf02622425_m1
BAX	
Caspase 3	Cf02622236_m1
Caspase 8	Cf02627553_m1
Caspase 9	Cf02627331_m1
**Housekeeping genes**	
ACTB	Cf04931159_m1
GAPDH	Cf04419463_gH
HPRT	Cf02690456_g1

Cell viability assay

The viability of MCA-B1 and DH82 cells when incubated with LPS was assessed using the 3-(4,5-dimethylthiazol-2-yl)-2,5-diphenyltetrazoliumcell cell viability assay (CyQUANT MTT), in accordance with the manufacturer's instructions. In brief, MCA-B1 or DH82 cells were seeded in 96-well cell culture plates at a density of 2×10^5 cells/mL and cultured overnight at 37 °C in an atmosphere of 5% CO2. The cells were then incubated with LPS (50 ng/mL) at 37 °C for 24 h. The culture media was then replaced with fresh media containing 1.2 mM MTT and incubated at 37 °C in an atmosphere of 5% CO2 for 2 h. The SDS-HCL solution was then added to each well and the plates further incubated at 37 °C in an atmosphere of 5% CO2 for 2 h. Absorbance (optical density, OD) was determined at 570 nm using a SpectraMax i3x microplate spectrophotometer (Molecular Devices, Berkshire, United Kingdom). Cells incubated with cell culture medium served as the "medium control". After coculture, 70% Ethanol was added in some of the medium control wells separately and served as "negative control". Cell viability was recorded as the OD570 absorbance in all wells.

Results

LPS (50 ng/mL) did not induce detectable cell death in canine MCA-B1 and DH82 cells

No significant effect of LPS on cell viability was observed in MCA-B1 cells following 24 h incubation (Figure S1A). The measured OD_{570} values were 2.6 ± 0.2 in the medium control and 2.1 ± 0.2 in LPS-treated cells, indicating no statistically significant difference. In contrast, ethanol treatment markedly induced cell death, as evidenced by a substantially reduced OD_{570} value (0.2 ± 0.0; $P < 0.0001$). A similar pattern was observed in DH82 macrophage-like cells, where LPS exposure did not significantly affect cell viability compared with the untreated control (Figure S1B).

(A)

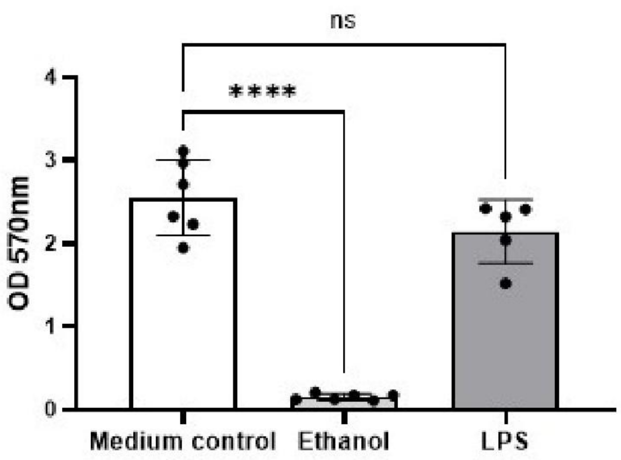

(B)

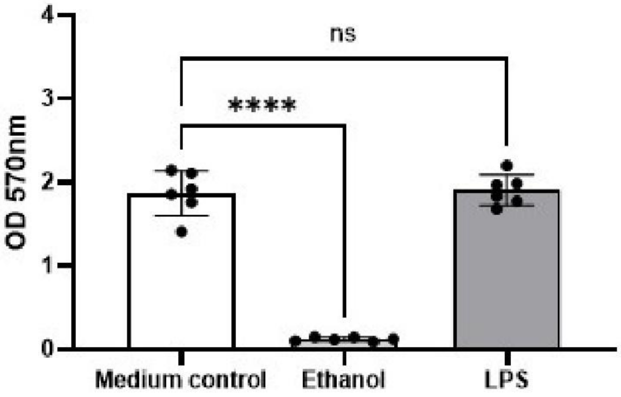

Figure S1. Effect of LPS on cell viability in MCA-B1 cells (A) and DH82 cells (B), measured by MTT assay. Data are expressed as OD_{570} values with standard error (SE) bars. The experiment was performed with six replicates per treatment group. ns, not statistically significant; ****, $P < 0.0001$.
